# Prolonged Jaundice Secondary to Amiodarone Use: A Case Report and Literature Review

**DOI:** 10.7759/cureus.3850

**Published:** 2019-01-08

**Authors:** Hunter Bratton, Mohammad Alomari, Laith A Al Momani, Tyler Aasen, Mark Young

**Affiliations:** 1 Internal Medicine, East Tennessee State University, Johnson City, USA; 2 Internal Medicine, Fairview Hospital, Cleveland, USA

**Keywords:** amiodarone, adverse effects, intrahepatic cholestasis, prolonged jaundice, hyperbilirubinemia

## Abstract

Adverse reactions to the antiarrhythmic medication amiodarone are severe, potentially life-threatening, and not rare. One in three patients on long-term therapy experience elevated liver enzymes, and clinically apparent liver toxicity occurs in 1% of patients treated. We report the case of a 76-year-old patient with amiodarone-induced intrahepatic cholestasis and prolonged hyperbilirubinemia despite the discontinuation of the offending agent. Current research hypothesizes that amiodarone leads to hepatic injury both by direct hepatotoxicity and by increasing the likelihood of hepatocytes to create abnormal, toxic metabolites. Increased awareness of such an adverse effect can guide clinicians toward the possible underlying etiologies of prolonged jaundice.

## Introduction

Amiodarone is used to manage life-threatening, irregular heart rhythms. It is on the World Health Organization’s (WHO's) Model List of Essential Medicines for being one of the most effective and safe medicines for addressing global public health needs. Despite its significant clinical benefits, amiodarone has been associated with many adverse effects. Between one-quarter and one-half of patients will discontinue amiodarone because of adverse effects, with 15% of patients experiencing a side effect within the first year and approximately half experiencing a side effect if used long-term [1]. Common side effects include corneal deposits, optic neuropathy, blue-gray skin discoloration, photosensitivity, hyperthyroidism, hypothyroidism, pulmonary toxicity, peripheral neuropathy, and liver function disturbances [2]. Amiodarone-induced hepatotoxicity is a rare adverse reaction, which may progress into hepatic fibrosis and cirrhosis [2]. In this report, we present the case of a 76-year-old patient with amiodarone-induced intrahepatic cholestasis with prolonged hyperbilirubinemia despite the discontinuation of the offending agent.

## Case presentation

A 76-year-old female patient presented to the hospital with a complaint of progressive jaundice of three weeks duration. The prior week, she experienced fatigue and malaise. Her past medical history was significant for colonic adenocarcinoma diagnosed 26 years prior to presentation, with a complicated course requiring small bowel resection and eventual total colectomy with end ileostomy. She had short gut syndrome, requiring total parenteral nutrition (TPN), paroxysmal atrial fibrillation, and a prior admission for sepsis.

She denied alcohol use, and her only medications were amiodarone, atorvastatin, and aspirin. On arrival, her vital signs were stable and a physical exam revealed marked jaundice along with mild right upper quadrant tenderness. She had a normal mental status with no asterixis.

Laboratory workup was remarkable for a total bilirubin of 26.7 mg/dL, direct bilirubin of 17.8 mg/dL, aspartate aminotransferase (AST) of 146 IU/L, alanine aminotransferase (ALT) of 74 IU/L, alkaline phosphatase of 99 IU/L, international normalized ratio (INR) of 1.4, platelet count of 68 K/uL, and albumin of 2.6 g/dL.

**Figure 1:**
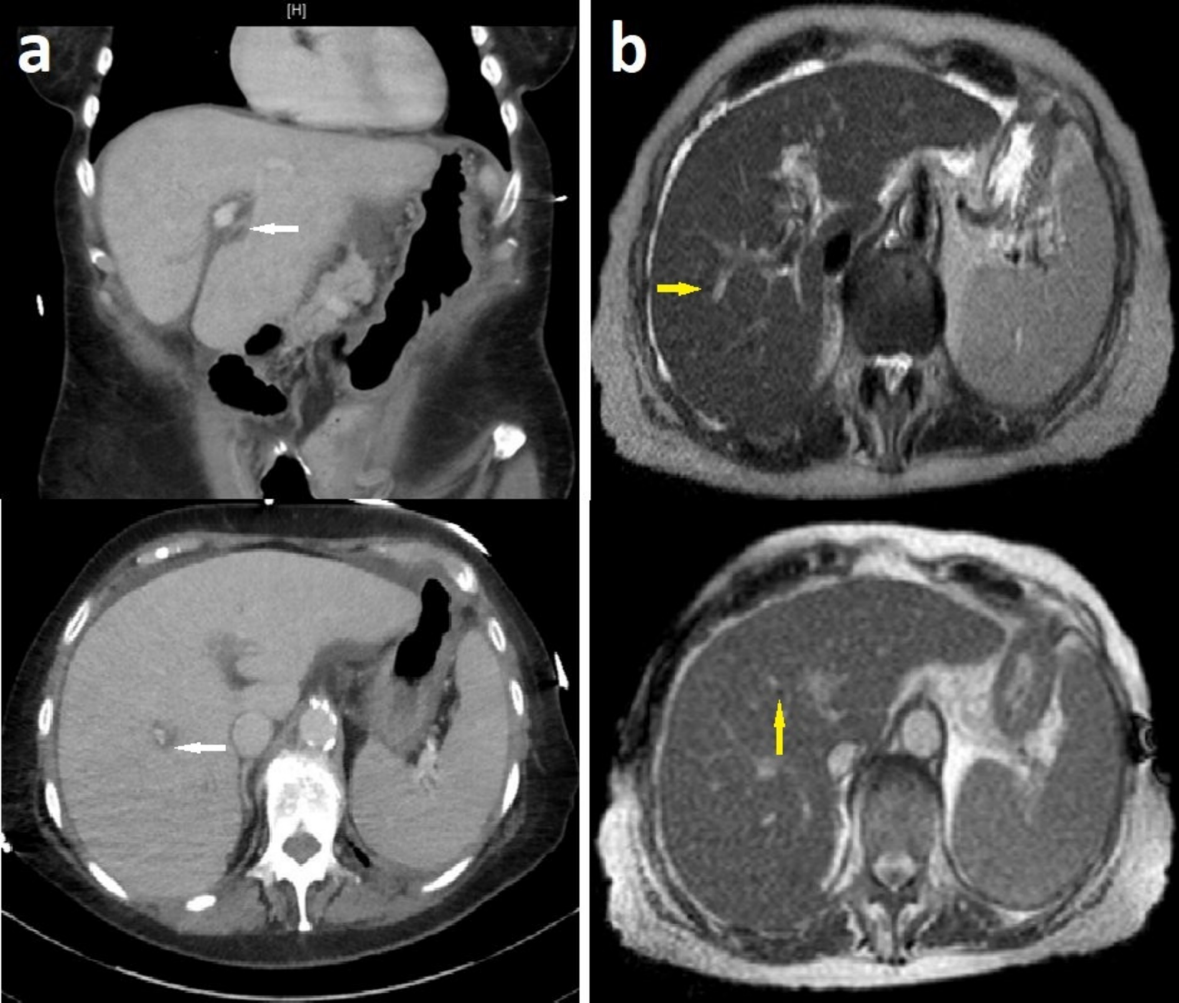
(a) Computed tomography of the liver (coronal and axial planes) showing periportal edema (white arrows); and (b) periportal halos (yellow arrows) on magnetic resonance imaging (T1 and T2-weighted images)

A computed tomography (CT) scan of the abdomen (Figure 1a) displayed periportal edema and a normal-appearing gallbladder and biliary tract. Abdominal ultrasound and magnetic resonance imaging (MRI) with cholangiopancreatography confirmed these findings (Figure 1b).

**Figure 2:**
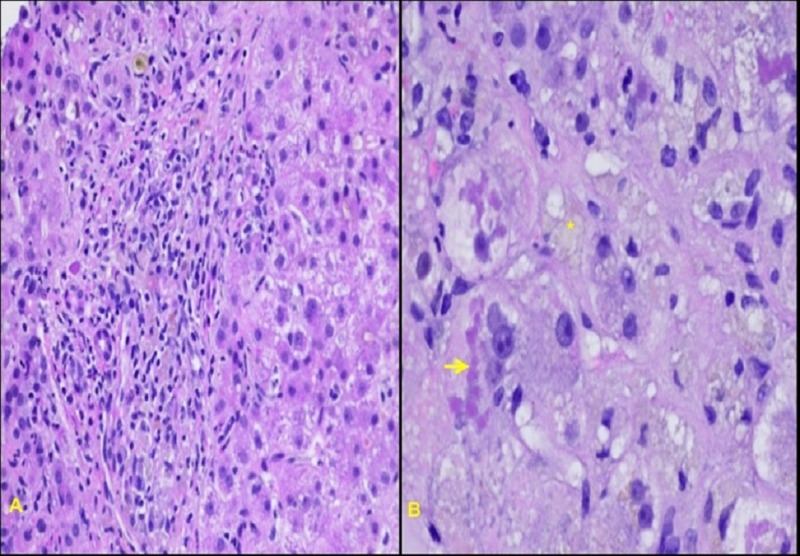
Biopsies of the liver: (A) histology notable for steatohepatitis-like ballooning degeneration, with (B) fatty change (asterisk) and prominent Mallory bodies (arrow), confirming amiodarone-induced hepatotoxicity

A thorough workup of common autoimmune, infectious, and genetic forms of liver diseases was negative. A liver biopsy was performed, and histology was most notable for steatohepatitis-like ballooning degeneration with prominent Mallory bodies, confirming amiodarone-induced hepatotoxicity (Figure 2).

## Discussion

Adverse reactions to amiodarone are severe and potentially life-threatening, making it vital for clinicians to be aware of the frequency and variability of adverse reactions. Having elevated liver enzymes is not a rare adverse reaction. Clinically apparent liver toxicity occurs in 1% of patients treated, with injury more common in patients treated with higher doses and prolonged therapy [3-4].

Hepatotoxicity varies widely from an asymptomatic, transient rise in serum aminotransferase levels to acute fulminant liver failure [5-6]. Approximately one in three patients on long-term therapy experiences serum enzyme elevation though, often, these elevations resolve without discontinuation [7]. Usually, the hepatotoxicity can be reversed with the discontinuation of amiodarone [8]. However, as in our patient, a rise in total bilirubin can be observed following the discontinuation of the medication, given its prolonged half-life of up to 100 days. A continued elevation in total bilirubin after medication discontinuation is indicative of a poor prognosis [9].

Current research hypothesizes that amiodarone leads to hepatic injury both by direct hepatotoxicity and by increasing the likelihood of hepatocytes to create abnormal, toxic metabolites [10]. Direct hepatotoxicity occurs because amiodarone is a potent inhibitor of phospholipase A [11]. Amiodarone complexes with phospholipids, causing the accumulation of lipophilic material in lysosomes and mitochondria as well as damage to the lipid bilayers of hepatocytes [12-13]. This damage leads to both macro- and micro-vesicular steatosis, as well as the characteristic Mallory body formation, fibrosis, foam cells, ductal proliferation, lipogranulomas, and ballooning degeneration seen on biopsy [14]. Similar in histopathologic appearance to alcoholic fatty liver disease, amiodarone-induced hepatotoxicity may be distinguished by the presence of phospholipid-laden lysosomal lamellar bodies (representing phospholipidosis) on an electron microscope [15]. If a CT scan is taken without contrast, the liver will often appear bright due to the accumulated iodinated medication [16].

Jaundice is a rare side effect and is likely the result of intrahepatic cholestasis [9,17]. This functional cholestasis leads to the retention of bile acids and bilirubin in the blood, to an elevation in alkaline phosphatase, and to the clinical manifestation of bilirubinostasis and jaundice [18]. Jaundice secondary to acute hepatic injury more often follows the intravenous infusions of amiodarone, and patients are often able to tolerate oral therapy without these complications [19-20]. Chronic injury occurs more often with long-term oral amiodarone therapy [19-20]. With our patient’s laboratory findings, the amiodarone prescription was switched to sotalol, another class III antiarrhythmic medication with a smaller adverse reaction profile. The patient’s generalized malaise gradually improved despite continued hyperbilirubinemia.

Liver injury caused by amiodarone can lead to liver failure and even death. Findings of hepatic fibrosis and necrosis correlate strongly with poor prognosis [9]. For this reason, periodic liver function testing every six months is indicated, with prompt discontinuation if there is more than a twofold elevation. No study has defined the relationship between the cumulative dose, duration of therapy, and overall toxicity of amiodarone; however, the cumulative dose likely corresponds with the overall toxicity. For this reason, maintenance doses should be minimized. Some animal studies have shown evidence that antioxidants such as N-acetylcysteine and vitamin E may have a protective effect against amiodarone-induced toxicity, although human case studies have not shown an improvement in liver function with such treatment [9]. To date, no approved therapies or antidotes exist to treat amiodarone toxicity; therefore, clinicians should be cognizant of the potential side effects of amiodarone and should stop therapy when amiodarone toxicity is suspected.

## Conclusions

While hepatotoxicity secondary to amiodarone use is well-studied, a literature review shows a very limited number of studies documenting amiodarone-associated hepatotoxicity leading to prolonged jaundice. In this report, we present the case of a 76-year-old patient with amiodarone-induced intrahepatic cholestasis with prolonged hyperbilirubinemia despite the discontinuation of the offending agent. Increased awareness of such an adverse effect and a thorough medical history can guide clinicians toward the possible underlying etiologies and an appropriate work-up for prolonged jaundice.
